# MFAP5 and TNNC1: Potential markers for predicting occult cervical lymphatic metastasis and prognosis in early stage tongue cancer

**DOI:** 10.18632/oncotarget.12446

**Published:** 2016-10-04

**Authors:** Xi Yang, Kailiu Wu, Siyi Li, Longwei Hu, Jing Han, Dongwang Zhu, Xuerui Tian, Wei Liu, Zhen Tian, Laiping Zhong, Ming Yan, Chenping Zhang, Zhiyuan Zhang

**Affiliations:** ^1^ Department of Oral & Maxillofacial - Head & Neck Oncology, Shanghai Key Laboratory of Stomatology, Shanghai Ninth People's Hospital, Shanghai Jiaotong University School of Medicine, Shanghai 200011, China; ^2^ Department of Oral Pathology, Shanghai Key Laboratory of Stomatology, Shanghai Ninth People's Hospital, Shanghai Jiaotong University School of Medicine, Shanghai 200011, China

**Keywords:** potential marker, metastasis and prognosis, tongue cancer, microarray

## Abstract

The purpose of this study is to identify candidate genes that could predict prognosis of early-stage tongue squamous cell carcinoma (TSCC) and its occult cervical lymphatic metastasis by large-scale gene expression profiling. Tumor tissue and matched normal mucosa samples were collected from patients with TSCC and analyzed with Affymetrix HTA2.0 high-density oligonucleotide array. Differentially expressed genes in TSCC with cervical lymph node metastasis (CLNM) were further analyzed with *Gene Ontology* and *Kyoto Encyclopedia of Genes and Genomes* for their functions and related pathways. A total of 107 differentially expressed genes (*p* < 0.05) were identified by microarray in TSCC samples with CLNM (n = 6) compared to those without CLNM (n = 6). Genes involved in the cell-matrix adherens junction and migration function including MFAP5, TNNC1, MGP, FBFBP1 and FBXO32 were selected and validated by RT-PCR in TSCC samples (*n* = 32). Of the five genes, MFAP5 and TNCC1 expressions were further validated by immohistochemistry (*n* = 61). The significant positive correlation between MFAP5 and TNNC1 expression (p<0.001) was observed. Notably, over-expression of MFAP5 and TNNC1 were correlated with CLNM, metastasis relapse-free survival and overall survival. Our findings indicated that MFAP5 and TNNC1 may be potential markers for predicting occult cervical lymphatic metastasis and prognosis of oral tongue carcinoma.

## INTRODUCTION

Tongue squamous cell carcinoma (TSCC) is the most common cancer in the oral cavity [1, 2], and surgery is the preferred treatment for primary tumors in TSCC patients [3, 4]. Although these primary tumors may be well controlled by surgical resection, a neck dissection is necessary when there is evidence of lymph node metastasis [5, 6]. However, there are no cancer cells in the cervical lymphatic tissues in some patients. Controversy exists in the selection of therapeutic strategy for TSCC, especially in N_0_ patients [7, 8]. Selective neck dissection may be appropriate in such cases. However, this prophylactic strategy may lead to higher morbidity and economic costs [2]. Even if neck dissection is not included in the management of early-stage TSCC, the mortality of N_0_ patients with occult lymphatic metastasis may also significantly increase [9]. Due to the lack of accurate and reliable methods for predicting occult cervical lymphatic metastasis, it is difficult to make treatment decisions in TSCC patients. This underscores the need to identify potential markers that can predict occult cervical lymphatic metastasis and prognosis of patients with early-stage TSCC.

The cellular and molecular heterogeneity of TSCC and the large number of genes potentially involved in oral carcinogenesis and progression emphasize the importance of studying multiple gene alterations on a global scale [10]. Gene expression analysis has proved to be a useful tool for predicting clinical outcome in human malignant tumors including head and neck cancers [11–14]. It also allows us to classify individual cancers and to better understand the molecular pathogenesis of cancers [10].

In this study, high-density oligonucleotide array was used to generate a molecular portrait of TSCC and to examine the correlations between gene expression patterns and clinically relevant parameters. We performed hierarchical clustering analysis and analyzed gene expression profiles by comparing primary tumors at the same T_2_ and N_0_ stage and their matched normal mucosa. Clinically significant genes were identified based on lymph node status and tumor stage. Data from the microarray analysis were validated by RT-PCR and immunochemistry, in which the specimens were obtained from our clinical research about treatment strategy of early-stage tongue cancer. This study aimed to identify candidate genes that could predict occult cervical lymphatic metastasis and prognosis of patients with early-stage TSCC.

## RESULTS

### Hierarchical clustering analysis

**Figure 1:**
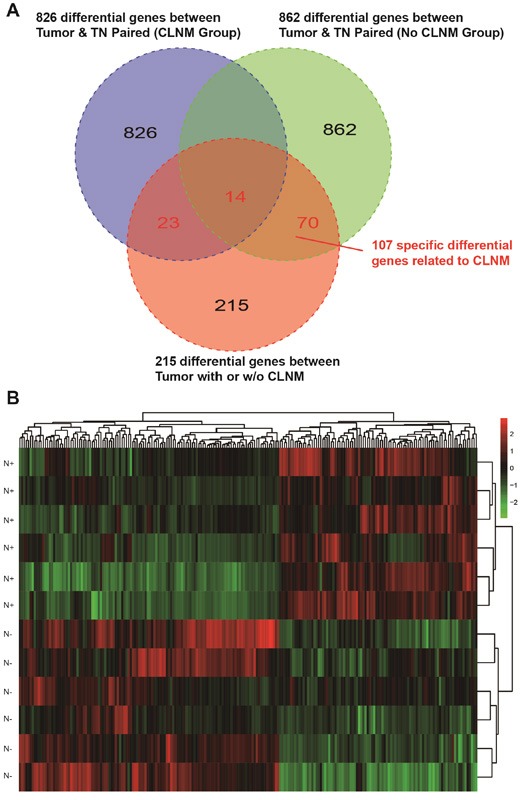
**A.** One hundred and seven differentially expressed genes in TSCC tissues with CLNM. Individual difference was removed by compassion of tumor tissues and matched normal tissues. **B.** Hierarchical clustering of gene expression data for TSCC with or without CLNM. It showed that 107 genes were up-regulated (red) or down-regulated (green) with over 2-fold change. **C.** MFAP5, TNNC1, MGP and other genes involved in cell adhesion, focal adhesion, cell-matrix adhesion and cell migration were present.

Gene expression profiles of 12 TSCC patients were analyzed by 67, 528 probes and the primary TSCC tumor samples were compared with their matched adjacent normal mucosa in the Affymetrix array. The clinical and pathological characteristics of all patients were shown in Supplementary Table S1. Clustering analyses were performed separately on (1) tumor samples and their matched normal tissue samples (TN paired, *n* = 12) and (2) tumor samples in two groups with or without cervical lymph node metastasis (CLNM) (*n* = 6 in each group). The genetic differences between these two groups were shown in Figure 1A. We found 826 genes in patients with CLNM and 862 genes in patients without CLNM that could distinguish tumors from matched normal tissues. In addition, 107 genes and their probes were significantly altered in patients with CLNM, 14 of which could be applied as indicators of tumor.

**Figure 2:**
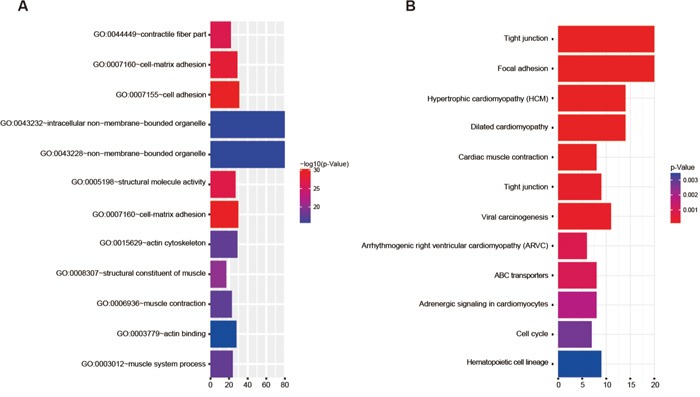
GO functional analysis and KEGG pathway analysis The target genes are concentrated in A. 12 major GO terms and B. 12 major KEGG pathways.

Figure 1B showed the 107 genes that were up-regulated or down-regulated along with the fold changes in gene expression, and Figure 2 showed the results of the GO functional analysis and the KEGG pathway analysis of these 107 genes. The pathways related to tight junction, focal adhesion, cell adhesion and cell-matrix adhesion were significantly altered in patients with CLNM. Therefore, genes involved in cell-matrix adherens junction and migration function (i.e., MFAP5, TNNC1, MGP, FBFBP1 and FBXO32) were selected for further analysis and validation.

### Quantitative real-time RT- PCR analysis

**Figure 3:**
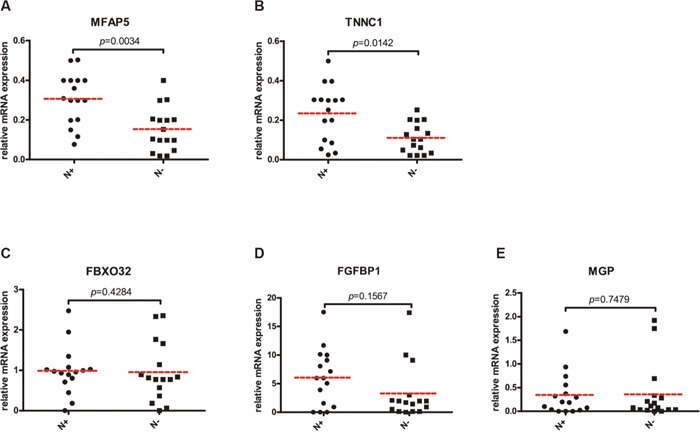
Quantitative comparison of MFAP5 (A), TNNC1 (B), FBXO32 (C), FGFBP1 (D) and MGP (E) mRNA expressions in 16 paired TSCC with or without occult CLNM *P* values were presented. N+: TSCC with occult CLNM; N-: TSCC without occult CLNM.

Specimens were collected from patients with Stages II (T_2_N_0_M_0_) TSCC for further analysis. Regression analysis was performed to compare clinical and pathological characteristics of patients without cervical nodal metastasis (N_0_) with those with nodal metastasis (Supplementary Table S2). We analyzed data from 12 patients whose tumor and matched normal mucosa were available for validating microarray results. Expressions of MFAP5, TNNC1, MGP, FBFBP1 and FBXO32 were analyzed in a larger cohort of 32 patients. We performed a two-step quantitative RT-PCR to validate expression changes identified by gene array analysis for the 5 selected genes in the 32 patients (Figure 3). Expressions of MFAP5 and TNNC1 were significantly elevated in N_0_ patients (MFAP5: *p* = 0.0034; TNNC1: *p* = 0.0142, respectively). FGFBP1 was also overexpressed, but with no significant difference between patients with and without CLNM (*p* = 0.1567).

### Immunohistochemistry of MFAP5 and TNNC1

**Figure 4:**
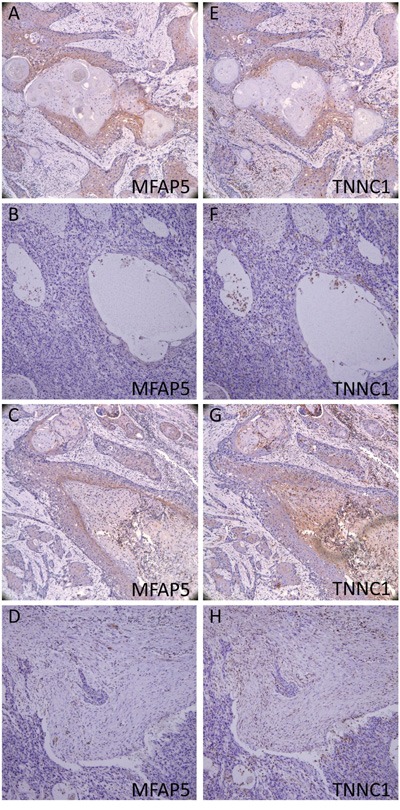
Immunohistochemistry of MFAP5 and TNNC1 **A. E.** strongly positive; **B. F**. Weak positive; **C. G**. Negative cancer cells; **D. H**. Negative CAFs.

Immunohistochemistry was performed to further investigate MFAP5 and TNNC1 expressions at the protein level. A total of 61 TSCC patients were identified with adequate histological material for immunohistochemistry and sufficient clinical data for survival analysis. Of these 61 patients, 56 were positive for MFAP5 and 57 were positive for TNNC1 (Figure 4). Table 1 showed MFAP5 and TNNC1 expressions and scores in patients with different cervical lymphatic conditions.

**Table 1:** MFAP5 and TNNC1 expression and scores

Group	N	MFAP5 Expression				MFAP5 Expression Rates	Mean MFAP5 Score	TNNC1 Expression				TNNC1 Expression Rates	Mean TNNC1 Score
		-	+	++	+++			-	+	++	+++		
pN-	27	4	8	8	7	25.20%	1.556	3	11	11	2	31.00%	1.481
pN+(w/o recurrence)	20	0	7	6	7	36.25%	2.55	0	5	9	6	44.50%	3.45
pN+(with recurrence)	14	1	4	3	6	37.50%	3	1	1	6	6	55.00%	4.429

### Survival analysis

**Figure 5:**
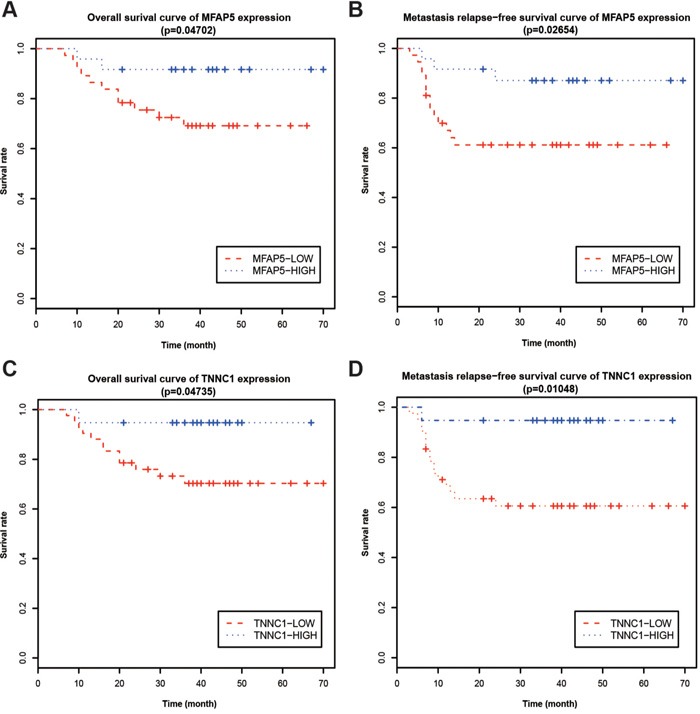
Kaplan-Meier survival analysis relative to MFAP5 and TNNC1 protein levels **A, C.** Overall survival rates and **B, D.** metastasis-free survival rates were analyzed for patients with either low or high MFAP5/TNNC1 expression.

The overall survival was 65.785 ± 2.902 months in patients negative for MFAP5 (*n* = 24), and 50.929 ± 3.840 months in patients positive for MFAP5 (*n* = 37); 50.929 ± 3.840 months in patients negative for TNNC1 (*n* = 19), 54.619 ± 3.782 months in patients positive for TNNC1 (*n* = 42); 62.929 ± 3.923 months in patients negative for both MFAP5 and TNNC1 (MFAP5- TNNC1-) (*n* = 14), 67.571 ± 2.340 months in patients positive for either MFAP5 or TNNC1 (MFAP5+TNNC1- or MFAP5-TNNC1+) (*n* = 15), and 48.516 ± 4.302 months in patients positive for both MFAP5 and TNNC1 (MFAP5+TNNC1+) (*n* = 32), respectively (Figure 5A). The overall survival of patients and factors affecting prognosis were shown in Table 2.

**Table 2:** Clinical information and factors related in prognosis

	Cases	Survival (month)	95%CI	*P-*value
**Gender**				0.227
Male	34	55.404±4.195	47.181-63.627	
Female	27	59.960±3.330	53.432-66.488	
**Age**				0.037
≤60y	36	47.857±3.600	40.802-54.913	
>60y	25	65.771±2.893	60.101-71.441	
**Pathological grades**				0.770
G1	13	40.092±2.703	34.794-45.391	
G2	45	57.954±3.393	51.305-64.604	
G3	3	49.333±13.608	22.661-76.006	
**Nerve and vascular invasion**				0.032
No	52	60.949±2.765	55.529-66.369	
Yes	9	42.333±9.223	24.256-60.410	
**Tumor status**				0.000
No recurrence	48	64.268±2.442	59.483-69.054	
Recurrence	13	30.923±4.992	21.139-40.707	
**N stage**				0.013
N0	27	63.537±2.397	58.839-68.236	
N1-2	34	51.835±4.514	42.988-60.683	
**Neck status**				0.000
CLNM-	27	63.537±2.397	58.839-68.236	
CLNM+(none of recurrence)	20	60.850±4.873	52.300-70.400	
CLNM+(recurrence after ND)	14	31.923±4.695	22.721-41.125	
**MFAP5 Scores**				0.047
-	24	65.785±2.902	60.096-71.474	
+	37	50.929±3.840	43.404-58.455	
**TNNC1 Scores**				0.047
-	19	64.000±2.920	58.277-69.723	
+	42	54.619±3.782	47.206-62.032	
**MFAP5 and TNNC1 Scores**				0.030
0	14	62.929±3.923	55.239-70.618	
+-	15	67.571±2.340	62.985-72.158	
++	32	48.516±4.302	40.084-56.947	

### Correlation between MFAP5 and TNNC1 expressions

There was a significant positive correlation between MFAP5 and TNNC1 expressions (Pearson correlation coefficient = 0.473, *p* < 0.001).MFAP5 and TNNC1 expressions were associated with CLNM and cervical lymphatic recurrence. A large majority of patients with CLNM were positive for MFAP5 (11/13) and TNNC1 (15/15) and had recurrence of TSCC. Positive MFAP5 and TNNC1 expressions were significantly related to TSCC recurrence (*p* = 0.006 and *p* = 0.004, respectively; Table 3). Patients with positive MFAP5 expression had a disease-free survival of 48.380 ± 4.428 months, which was significantly shorter than that in patients with negative MFAP5 expression (65.171 ± 3.278 months).

**Table 3:** Cox regression of prognostic risk in TSCC patients

Factor	Cox regression		Cox regression	
	Univariate	*p*	Multivariate	*p*
Age(≤60years)	4.319(0.957-19.499)	0.057	4.562(0.994-20.944)	0.050
Nerve vascular invasion	3.363(1.032-10.954)	0.044	5.107(1.445-18.054)	0.011
Co-expression of MFAP5 and TNNC1	5.969(1.320-26.991)	0.020	7.854(1.640-37.621)	0.010

To assess the influence of each factor, univariate and multivariate analysis was performed to assess which factors remained independent indicators of prognosis. On multivariate analysis, the adjusted HR for prognosis was 7.854 for co-expression of MFAP5 and TNNC1 expression. In addition, the prognostic factors of age(≤60years) and nerve vascular invasion was still significant, but inferior to prognostic of both proteins co-expression.(Table 4)

**Table 4:** Correlation between MFAP5 and TNNC1 expression

	CLNM-	CLNM+ (w/o recur)	CLNM+ (with recur)	*P*	CLNM-	CLNM+	*P*
MFAP5-	17	5	2	0.006	17	7	0.002
MFAP5+	11	15	11		11	26	
TNNC1-	14	5	0	0.004	14	5	0.003
TNNC1+	14	15	13		14	28	
MFAP5-TNNC1-	11	3	0	0.006	11	3	0.002
MFAP5+TNNC1-/MFAP-TNNC1+	9	4	2		9	6	
MFAP5+TNNC1+	8	13	11		8	24	

CLNM = cervical lymphatic neck metastasis

## DISCUSSION

Molecular biomarkers involved in the pathogenesis of head and neck cancers including oral cancer have long been a subject of intense research. Abnormal expressions of MAGED4B, KLK13, MMP9 and GLUT3 were correlated with CLNM [10, 15, 16]. However, TSCC at early stage and its occult cervical lymphatic metastasis have rarely been investigated. In clinical practice, it remains controversial whether selective neck dissection is needed in the treatment of TSCC, especially in patients with cT_2_N_0_M_0_ tongue cancer. Thus, we performed transcriptome profiling of cT_2_ tongue cancer at the same TNM stage. One group had lymph node metastasis within the 6-month follow-up and another group had no metastasis in the following 18 months.

We have focused on adhesion molecules (including focal adhesion, junction adhesion, etc.) and cell migration based on the GO analysis and the KEGG analysis. MFAP5, TNNC1, MGP, FGFBP1 and FBXO32 genes were selected for validation using real-time PCR. Some molecules, like ZEB1, which played an important part in EMT process, were significantly changed in TSCC. MFAP5 and TNNC1 showed accordance between real-time PCR and Affymetrix microarray, which were further validated by immunochemistry using specimens from our clinical trial.

MFAP5 (also known as microfibril-associated glycoprotein 2, MAGP2) is a multifunctional protein that plays an important role in elastic microfibril assembly and modulating endothelial cell behavior, and thus it is considered as a novel modulator in cell survival [17]. MFAP5 expression was increased in head, neck, pancreatic, lung, and ovarian cancers [18–21]. However, the role of MFAP5 in these cancers remains to be elucidated. MFAP5 was an independent predictor of survival in advanced ovarian cancer, and could promote tumor proliferation and endothelial cell motility through αβV_3_ integrin mediated signaling, providing a potential mechanistic link between MFAP5 and angiogenesis as well as patient survival [22, 23]. In this study, MFAP5 had a higher correlation with occult cervical metastasis than other genes examined, suggesting the potential role of MFAP5 in the diagnosis of occult cervical metastasis. In addition, the mRNA and protein levels of MFAP5 were significantly elevated in patients with poor prognosis, thus indicating that MFAP5 could be an independent prognostic marker for TSCC and its occult cervical metastasis.

TNNC1 is known as a Ca^2+^-binding subunit that can facilitate the relationship between actin and myosin in muscle cells. However, in non-muscle cells, TNNC1 may act as a regulatory protein for cellular locomotion, cytoplasmic streaming and cytokinesis, rather than as a structural protein [24]. TNNC1 was over-expressed in ovarian cancer cells, and elevated TNNC1 expression regulated epithelial cancer cell motility and invasion potential via cytoskeleton reorganization [25, 26]. TNNC1 was an effector protein of MFAP5 in stomata, which promoted cell motility and invasion potential via tumor-stroma crosstalk and subsequently affected clinical outcomes [21]. However, the relationship between MFAP5 and TNNC1 in oral cancers remains unclear. The TNNC1 expression was altered in oral cancer. Our results showed that MFAP5 and TNNC1 expressions were associated with CLNM and cervical lymphatic recurrence. A majority of patients with CLNM were positive for MFAP5 (11/13) and TNNC1 (15/15). Thus, TNNC1 appeared to be more sensitive than MFAP5 in predicting the prognosis of TSCC and its occult cervical lymphatic metastasis. However, further studies are needed to validate our results due to limited sample size and detection methods used.

In conclusion, MFAP5 and TNNC1 could be potential markers for predicting occult cervical lymphatic metastasis and prognosis of oral tongue carcinoma.

## MATERIALS AND METHODS

### Sample selection

Fresh tumor and normal tissue specimens were collected from 12 patients underwent surgical resection for TSCC and selective neck dissection at the Department of Oral & Maxillofacial-Head and Neck Oncology, Shanghai Ninth People's Hospital, School of Medicine, Shanghai Jiaotong University, from July 2013 to August 2014 following the guidelines established by our university. All patients gave their informed written consent to participate in this study. Tumor specimens for PCR and immunochemistry were selected by computer-generated random numbers from 171 patients enrolled in a previous prospective randomized trial [27]. Adjuvant treatment was given after operation. In each patient, TSCC samples were collected near the forward edge of the tumor instead of the necrotic center, and then immediately snap frozen and stored in liquid nitrogen until use. Histologically normal mucosa was obtained 3 cm away from the tumor in all cases.

Tumors were staged as T_2_ according to the AJCC/UICC TNM classification (7th edition). In this study, node-positive refers to the presence of positive CLNM, while node negative refers to the absence of positive CLNM for at least 18 months after operation based on histological diagnosis after neck dissection.

### Transcriptome microarray and functional analysis

Tumor and normal tissues obtained from 12 patients were used for oligonucleotide microarray analysis. Twenty (TN paired) patients had primary tumor samples and matched normal mucosa available for analysis, which were used for oligonucleotide microarray analysis. Total RNA was extracted from snap-frozen tissue samples following the manufacturer's protocol and re-purified by RNA easy Mini-spin column (Qiagen). The cDNA was used for *in vitro* transcription amplification in the presence of biotinylated nucleotides. The labeled cRNA was fragmented and then hybridized against the GeneChip Human Transcriptome Array 2.0 oligonucleotide arrays (Affymetrix, Santa Clara, CA). The arrays were scanned using a Hewlett Packard confocal laser scanner and analyzed with MicroArray Suite 5.0 (Affymetrix). Differentially expressed genes were further analyzed with *Gene Ontology (Go)* and *Kyoto Encyclopedia of Genes and Genomes (KEGG)* for their functions and related pathways.

### RNA preparation and real-time RT-PCR

RT-PCR was performed for the expression of MFAP5, MGP, FGFBP1, FBXO32 and TNNC1 in a larger cohort of 32 patients. Two μg of total RNA was reverse transcribed with MultiScribe™ Reverse Transcriptase (Applied Biosystems, Inc., Foster City, CA). Gene specific primers were designed using the Primer3 Program. The PCR primer sets (in 5'-3' direction) were as follows: MFAP5 forward: GCCAGCCAAAGTAGGAACAG, MFAP5 backward: AGCAAGAAACAGCAGCACCT; MGP forward: CCCTCTCAACTGCTCTGGTT, MGP backward: CAGGCTCTTCATGGTTTCGT; FGFBP1 forward: CCCTGCTCTCCTTCCTCCTA, FGFBP1 backward: GTGTTGCCCAGAGTGTCCTT; FBXO32 forward: AGCGGATGTTCATTCTCCAC, FBXO32 backward: AAATGCCCAGCAGACAAAGT; TNNC1 forward: CAGCAAAGGGAAATCTGAGG, TNNC1 backward: TGATGGTCTCGCCTGTAGC.

### Immunohistochemistry

The expression levels of MFAP5 and TNNC1 proteins in TSCC were further examined by immunohistochemistry. The TSCC tissues were embedded and cut into 5-μm sections. Then, the sections were stained by monoclonal antibody to TNNC1 (1: 100, WH0007134M1, Sigma-Aldrich) and polyclonal antibody to MFAP5 (1: 100, HPA010553, Sigma-Aldrich), and examined by two independent pathologists in our hospital. MFAP5 and TNNC1 expressions were examined using a scoring method. The mean percentage of positive tumor cells was determined by examining 500 cells in at least 5 sections at 40x10 magnification. Cells were assigned to one of the following four classes according to the percentage of positive cells (PP): 0) ≤ 24%; 1) 25-49%; 2) 50-74%; and 3) 75-100%. The intensity of MFAP5 and TNNC1 staining (SI) was scored as: 0) no, -; 1) weak, +; 2) moderate, ++; and 3) intense, +++. The final immunoreactive score was calculated by the following formula: IRS = SI × PP. A score higher than 0 indicates positive expression, whereas a score lower than 0 indicates negative expression. The stained tissues were scored by researchers who were blind to the patients.

### Statistical analysis

The Kaplan–Meier method was used to estimate metastasis-free rates using SPSS version 13.0 (IBM, Armonk, NY), and the χ^2^ analysis and Mantel-Haenszel log-rank test were used to calculate the statistical significance (P-value) of the difference between the curves. *P* < 0.05 was considered as statistically significant. The follow-up period was defined as the interval from the time of biopsy or dissection for pathological diagnosis to the final follow-up date of January 2016. The neck nodal metastasis was defined as positive cervical lymph node when the patient underwent neck dissection in one stage or neck metastasis during the wait-and-see period. The overall and disease-free survival was determined by using the Kaplan-Meier method and the Log-rank test.

## SUPPLEMENTARY MATERIALS TABLES


