# Supplementary material for: MFAP5 and TNNC1: Potential markers for predicting occult cervical lymphatic metastasis and prognosis in early stage tongue cancer
Source: Oncotarget. 2016 Oct 4;8(2):2525–35. doi: 10.18632/oncotarget.12446 (PMC5356821; doi:10.18632/oncotarget.12446)
Supplement: Supplementary file 1 [file oncotarget-08-2525-s001.pdf]

MFAP5 and TNNC1: Potential markers for predicting occult cervical lymphatic metastasis and prognosis in early stage tongue cancer

SUPPLEMENTARY TABLES

Supplementary Table S1: Patient clinical information and histological date for tissue sample and matched normal mucosa for microarray analysis

Patients	Gender	Age	Surgery	T stage, size	N stage, level	Invasion depth	Pathological grade
1	male	39	Resection + SOND + SIF	T2,2.5*2.0m	N0	10mm	I-II
2	male	48	Resection + SOND + RFFF	T2,3.5*3.0cm	N0	12mm	I
3	female	72	Resection + SOND + SIF	T2,3.8*3.0cm	N0	8mm	I-II
4	male	55	Resection + SOND + RFFF	T2,3.0*2.5cm	N0	6mm	I-II
5	female	51	Resection + SOND + RFFF	T2,3.9*2.5cm	N0	10mm	II
6	male	66	Resection + SOND + RFFF	T2,3.0*3cm	N0	7mm	II
7	female	67	Resection + SOND	T2,2.1*0.8cm	N1,Level I1/5(+)	3mm	I
8	female	66	Resection + SOND	T2,2.0*2.0cm	N1,Level II 1/5(+)	4mm	I-II
9	male	47	Resection + SOND + RFFF	T2,3.0*2.ocm	N2,Level I 1/7,II 1/5,III 1/17 (+)	8mm	I-II
10	male	57	Resection + SOND + RFFF	T2,3.0*3.0cm	N2,Level I 2/4,II 2/7 (+)	7mm	II
11	male	64	Resection + SOND + NIF	T2,3.1*1.5cm	N2,Level II 2/4,III 1/5 (+)	9mm	II
12	female	40	Resection + SOND + RFFF	T2,3.8*2.9cm	N2,Level I 1/4,II 1/7 (+),ECS	10mm	II-III

SOND = Supraomohyoid neck dissection; SIF = Submental island flap; NIF = Nasolabial island flap; RFFF = Radial forearm free flap

Supplementary Table S2: Clinical-pathological characteristic of T2 patients in validation group

Parameter	Validation	
	CLNM+	CLNM-
Total	16	16
Age (yrs)		
Median (Range)	61.1 (35-80)	54 (33-81)
Gender		
Male	11	12
Female	5	4
Smoking history		
Yes	9	8
No	7	8
Alcohol history		
Yes	6	7
No	10	9
Histological grading		
Grade I	2	3
Grade II	11	12
Grade III	3	1
Pathological lymph node involvement		
N0	0	16
N1	3	0
N2	13	0
N3	0	0

CLNM = Cervical lymph node metastasis
